# Recent Challenges and Trends of Polyhydroxyalkanoate Production by Extremophilic Bacteria Using Renewable Feedstocks

**DOI:** 10.3390/polym15224385

**Published:** 2023-11-11

**Authors:** Justyna Możejko-Ciesielska, Subhasree Ray, Shivangi Sankhyan

**Affiliations:** 1 Department of Microbiology and Mycology, Faculty of Biology and Biotechnology, University of Warmia and Mazury in Olsztyn, 10719 Olsztyn, Poland; 2 Department of Life Sciences, School of Basic Science and Research, Sharda University, Greater Noida 201310, India; shivangisankhyan21@gmail.com

**Keywords:** biopolymers, extremophiles, polyhydroxyalkanoates, copolymers

## Abstract

Polyhydroxyalkanoates (PHAs) are biodegradable polymers with immense potential in addressing the global plastic pollution crisis and advancing sustainable bioplastics production. Among the various microbes known for PHA production, extremophilic bacteria possess unique capabilities to thrive under extreme conditions, making them attractive candidates for PHA synthesis. Furthermore, the utilization of renewable feedstocks for PHA production aligns with the growing demand for sustainable bioplastic alternatives. A diverse range of extremophilic bacteria, especially halophiles and thermophiles, has provided cost-competitive platforms for producing customized PHA polymers. Extremophilic bacteria offer unique advantages over mesophiles due to their contamination resistance, high cell density growth, and unique culture conditions. The current status of *Halomonas* spp. as a chassis further allows exploration of metabolic engineering approaches to overcome the challenges associated with current industrial biotechnology. This article especially focuses on extremophilic bacteria and explores recent advances in utilizing renewable feedstocks such as lignocellulosic biomass, agro-industrial residues, and waste streams for PHA production. The integration of biorefinery concepts and circular economy principles in PHA manufacturing is also examined. This review is an attempt to provide an understanding of renewable substrates as feedstocks and emerging trends in PHA production by extremophilic bacteria. It underscores the pivotal role of extremophiles and sustainable feedstock sources in advancing the feasibility and eco-friendliness of PHAs as a promising biopolymer alternative.

## 1. Introduction

Conventional plastics are versatile, durable, and relatively inexpensive to produce, which has led to their widespread use in various industries and everyday products. While petroleum-based plastics have many advantages, they also pose significant environmental challenges. Their production based on nonrenewable fossil resources causes the depletion of fossil fuel, microplastic release, and greenhouse emissions. Moreover, most of them persist in the environment for hundreds of years, leading to plastic pollution. Improper disposal and recycling practices have resulted in the accumulation of plastic waste in landfills, oceans, and other ecosystems, causing harm to wildlife and the environment. Therefore, many efforts are being made to develop more sustainable alternatives to conventional plastics, such as biodegradable plastics made from renewable resources [1]. According to the latest market data provided by “European Bioplastics”, bioplastics account for about one percent of the more than 390 million tonnes of plastic produced annually (https://www.european-bioplastics.org/market/, accessed on 2 October 2023). The global production capacity of biodegradable plastics is estimated to increase from around 2.2 million tonnes in 2022 to only approximately 6.3 million tonnes in 2027 (https://www.european-bioplastics.org/market/, accessed on 2 October 2023). Hence, there is a need to develop strategies for the production of eco-friendly biopolymers. There are several alternatives to synthetic plastics. Among them, starch-based composite materials, polylactic acid, and polycaprolactone are commonly produced and used for specific purposes. However, it must be noted that not all the biodegradable polymers are of biological origin and vice versa. Polyhydroxyalkanoates (PHAs) are the only polyester of biological origin that has wider physico-chemical and thermoplastic properties similar to synthetic plastics. These biopolymers are of microbial origin, biodegradable, and compostable in the natural environment, and therefore have attracted much attention globally.

Microbial polyhydroxyalkanoates (PHAs) are a group of polyesters which have similar properties to conventional polymers; however, they are a greener alternative to them. They are produced intracellularly as distinct granules by numerous bacteria. PHA monomers are grouped into three categories: short chain length (scl-PHA), which consist of monomers containing 4 to 5 carbon atoms; medium chain length (mcl-PHA), containing 6 to 14 carbon atoms; and long chain length, with more than 15 carbon atoms [2]. Too-high production costs still represent a major barrier to customizing PHAs on a large scale. In order to develop a successful bioprocess of PHA production, it is necessary to consider the optimization of upstream and downstream processes, as they are interconnected and influence the overall economics [3]. The PHA productivity, composition, and properties are dependent on several factors, such as the carbon source used, the bacterial producer, and the growth media composition, a type of cultivation and biopolymer recovery method [4].

Microbial processes have two advantages over synthetic chemistry in terms of biopolymer production: first, they can use renewable carbon sources as substrates, and second, the range and specificity of molecules produced biologically are greater than those produced by synthetic chemistry [5]. In the past few years, extremophilic bacteria have gained significant attention in the field of eco-friendly green biopolymer production due to their unique abilities and features [6]. Importantly, these microorganisms can grow on low-cost culture media supplemented with industrial waste or surplus feedstocks. Even the application of saline sea water to eliminate the usage of fresh water and salt supply is possible in the fermentation processes of extremely halophilic bacteria [7]. A novel concept of “next generation industrial biotechnology” created by Chen and Jiang [8] establishes the use of extremophilic bacterial strains as a solution for improving efficiency, sustainability, and scalability in biotechnological processes. PHA production has been described for various extremophiles, but for downstream advantages, extremophilic bacteria are considered suitable [9]. Due to high PHA production costs, numerous attempts have been made to optimize cultures supplemented with waste feedstock [10,11]. However, PHA production by extremophilic bacteria from renewable resources is the subject of very few studies.

This review focuses on the current state of knowledge of PHA production by extremophilic bacteria, with special attention on their potential to utilize waste feedstocks, and also provides challenges and future research directions in their synthesis.

## 2. Bacteria from Extreme Habitats as PHA Producers

Bacteria are remarkably adaptable microorganisms that have evolved to survive and thrive in a wide range of extreme conditions. Their ability to adapt quickly to changing environments is a result of their genetic flexibility and various survival mechanisms. This adaptability has enabled bacteria to colonize every corner of the earth, showcasing their remarkable resilience and evolutionary success [12]. Some of the extreme conditions to which bacteria have adapted include high temperatures, low temperatures, high pressure, high salinity, acidity or alkalinity, radiation, and extreme dryness. Such bacteria open an interesting area of producing value-added metabolites and they possess the advantages of conducting such processes.

**Figure 1:**
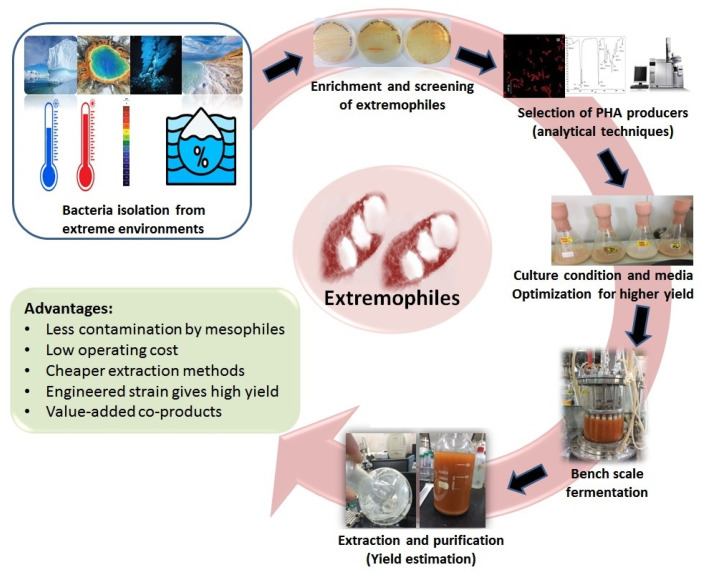
Bacteria from extreme niches as PHA producers.

Microorganisms from extreme habitats have shown remarkable capabilities in producing PHAs. Their ability to synthesize PHAs allows them to adapt to the challenging conditions of their environments, making them valuable candidates for biotechnological applications and sustainable bioplastics production. Additionally, studying PHA production in extremophiles can provide insights into the mechanisms of stress tolerance and resource management in these unique microorganisms (Figure 1). The adaptation to harsh habitats is driven by stress response metabolites and genes [9]. Overall, the accumulation of PHAs in the cells of extremophilic bacteria aids in their adaptation to stressful conditions, providing a reserve of carbon and energy, and offering protection to the cells. This enables extremophiles to survive and thrive in environments with extreme conditions, where many other microorganisms would struggle to survive.

In comparison with mesophilic bioprocesses, extremophilic cultivations are suggested to be energy-efficient. Furthermore, the reduced risk of contamination by mesophilic microorganisms is one of the advantages of extremophilic fermentation processes. The bioprocess can be operated under non-sterile growth conditions [13]. However, the high production costs of PHA compared with other (bio)polymers still limits their further expansion in the market. Therefore, strong attempts are currently targeted on searching for novel PHA producers that would grow on low-cost carbon sources and increase the economical feasibility and competitiveness of industrial PHA production. There are several extremophiles (especially halophiles and thermophiles) known and explored for large-scale PHA production. Among the well-known halophilic bacteria, *Halomonas* spp. and *Paracoccus* spp. are the most important halophiles studied in detail [14,15,16]. In comparison, other extremophiles are less explored for PHA production. Few thermophiles, such as *Aneurinibacillus* sp. H1, *Bacillus shackletonii*, *Schlegelella thermodepolymerans*, *Caldimonas taiwanensis*, *Geobacillus* spp., and *Chelatococcus thermostellatus*, are reported for PHA production with an advantage over halophiles during fermentation, i.e., reduced corrosion [17,18,19,20].

*Colwellia psychrerythraea* 34H was observed to possess the PHA biosynthetic genes, though PHA production was not reported [21]. Later, other related deep-sea bacteria belonging to *Colwellia* spp., *Shewanella* spp., and *Moritella* spp. were characterized for PHA and oligo-hydroxyalkanoate synthesis on fructose, glucose, gluconate, and plant oils as the sole source of carbon [22]. A large-scale screening study was also conducted to identify PHA producers from polar regions. Hundreds of bacterial isolates from 25 different habitats of both polar regions were screened based on agar-based tests, staining-microscopy, and genetic methods. The rich diversity of PHA-producing psychrophiles was obtained, mostly belonging to the *Comamonadaceae*, *Microbacteriaceae*, and *Micrococcaceae* families, highlighting that there are many more microbes to be tapped from extreme environments [23]. Interestingly, *Iodobacter* sp. PCH194, isolated from the Himalayan region, was found to be a eurypsychrophile, having the potential to co-produce violacein along with PHA [24]. Polyextremophiles are rarely reported for PHA production. Recently, *Halomonas* sp. strain 363 and *Paracoccus* sp. strain 392, found at Southern Ocean sea ice, were shown to accumulate PHA at temperatures as low as 4 °C [25].

## 3. Effects of Renewable Feedstocks on PHA Production by Extremophiles

**Figure 2:**
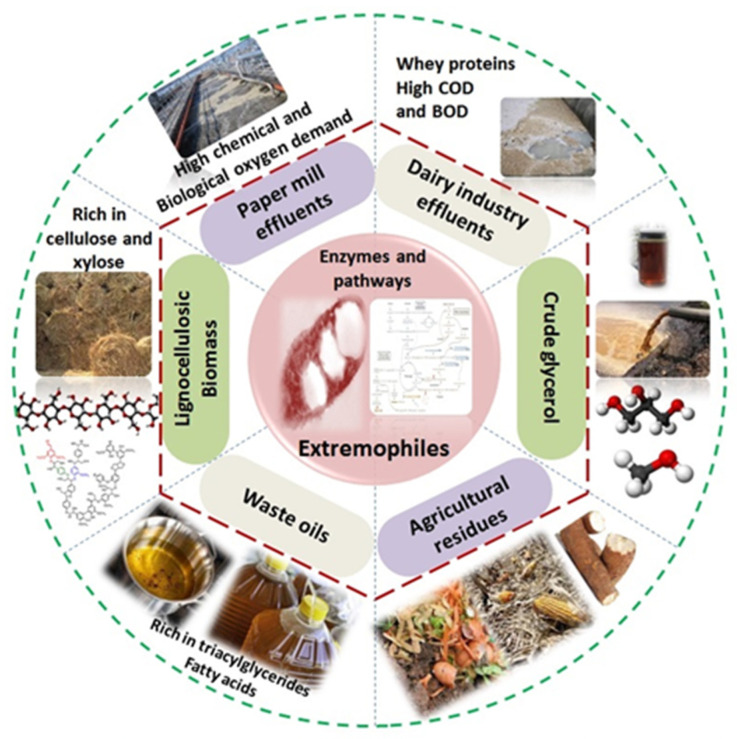
Renewable feedstocks on PHA production by extremophiles.

Extremophiles are capable of utilizing a broad range of carbonaceous substrates for their growth and metabolism (Figure 2). Production of PHA using refined or pure sugar substrates leads to an increase in overall production cost (approximately 30–50%). Thus, the use of renewable feedstocks may reduce the overall cost, provided that the processing of such biomass to generate simple sugars should not be complex and/or expensive. Few studies have shown PHA production by extremophiles fed on renewable feedstocks such as those from agricultural wastes and industrial wastes. Among them, spent cooking oils, crude glycerol, and cheese whey are some of the important and low-cost substrates that come from various industries (Table 1). In addition, it has been argued that the use of methane by thermophilic methanotrophs results in a reduction of up to 22% in PHA production cost [26]. Other C1 carbon sources such as CO_2_ can also be used for PHA production [27]. A techno-economic analysis based on PHA production at a scale of 100 thousand tonnes per annum revealed that the PHA cost is reduced from 4.1 USD/kg to 3.2 USD/kg [28]. Several studies have focused on diverting renewable resources and carbon-rich industrial effluents to lower the PHA production cost, using pure or mixed microbial cultures.

### 3.1. Waste Oils

Several extremophiles, mostly halophiles, were found to use waste oils and lipid-rich substrate for PHA production. Among the most notable bacterial species, *Halomonas hydrothermalis* and *Halomonas neptunia* were capable of synthesizing PHA copolymer with an HV content of up to 50.15 mol % [32]. Supplementation of mild surfactant such as Tween-80 helps in the oil dispersion and facilitates its consumption by microbes. *Paracoccus* sp. LL1 effectively consumes waste frying oil in the presence of Tween-80 to produce PHA copolymer of up to 30.89% of its total biomass, along with valuable pigments [16]. However, the PHA yield of *Paracoccus* sp. was relatively lower than other halophilic species (Table 1). Another halophile belonging to genus *Salinivibrio* spp. grows very well using waste fish oil supplemented with glycerol as carbon sources. Under fed-batch culture, a very high biomass of 69.1 g/L (51.5% PHA copolymer) was obtained within 78 h of cultivation [31].

### 3.2. Crude Glycerol

Crude glycerol is readily being generated as a byproduct of the biodiesel industry during the transesterification reaction. The effluents from these industries are rich in glycerol content (up to 70% glycerol), with other minor contaminants. After minor pretreatment, or sometimes untreated, effluents can be directly used as a feed for the generation of various bioproducts, including PHA. Both halophilic and thermotolerant microbes are known to grow on crude glycerol and produce PHA in varying quantities. Among them, *Halomonas* spp. and *Paracoccus* spp. were explored in detail. A marine isolate, *Halomonas hydrothermalis* SM-P-3M is among the highest P(3HB) producer in terms of accumulation per unit biomass (Table 1). The isolate could grow on minimal media supplemented with glycerol-rich jatropha biodiesel byproduct and produce P(3HB) up to 75% of its total biomass [35]. Compared to *Halomonas* spp., *Paracoccus* spp. produces PHA up to 40% of cell dry mass (CDM). Yet, both strains have been found to co-produce pigments along with the intracellular polymer accumulation [15]. Co-production of high-value compounds along with PHA, especially the extracellular co-product, is considered to be favorable for economic production of biopolymers [41]. *Pseudomonas* spp. are a well-known producer of mcl-PHA having unique properties. Satoh et al. [34] identified a unique thermotolerant isolate, *Pseudomonas* sp. strain SG4502, that can grow at high temperatures (45 °C was optimal for PHA production) and utilize biodiesel fuel by-products. Biodiesel fuel by-product largely consists of glycerol, which was used as a carbon source by *Pseudomonas* sp. to accumulate up to 40.6% of PHA in CDM (Table 1). Composting sites are good source of thermo-tolerant microbes. Recently, a thermotolerant PHA producer identified as *Cupriavidus* sp. CB15 was isolated from corncob compost [42]. This natural isolate showed very high accumulation (PHB content of 75.3 wt % of CDM) using glycerol as a carbon source, which was further optimized to achieve a highest yield of 74.4 wt % of CDM with a 2.12-fold increase compared with unoptimized conditions.

### 3.3. Cheese Whey and Cheese Whey Mother Liquor

Cheese whey is another such readily available sugar-/protein-rich source obtained from the dairy industry. However, pretreatment of cheese whey is sometimes required. Compared with chemical hydrolysis, enzymatic hydrolysis of cheese whey was found to be suitable for higher PHA yield. It was found that enzymatic hydrolysate of cheese whey leads to increased PHA yield with higher HV mol % within the copolymer [37,43]. Cheese whey and whey mother liquor were also used as feed to grow *Paracoccus homiensis* during the PHA production process. Mineral salt medium with or without nitrogen limitation was tested at 40 g/L to 70 g/L of cheese whey mother liquor or cheese whey supplementation. The highest PHA content was found with cheese whey mother liquor (Table 1). This was relatively suitable substrate (with or without nitrogen limitation) compared to cheese whey. The latter resulted in a maximum of 25% PHA content under non-limiting nitrogen supplementation [37]. Similarly, thermophilic bacteria *Thermus thermophilus* HB8 can grow on whey-based media to produce a novel PHA heteropolymer consisting of scl-mcl monomers [36]. Here, a maximum PHA yield of 35% was obtained (Table 1). For large-scale operations, just like halophiles, thermophiles keep contaminants at bay and are favorable for lower operational costs towards cooling.

### 3.4. Other Waste Materials as Substrate

Halotolerant microbes such as *Halomonas* spp. are favourable for industrial use as they grow in a saline environment unsuitable for other microbes, eliminating the need of sterilization or aseptic fermentation processes. Another advantage of halophilic microbes is their inherent tendency to lyse in a hypotonic solution, simplifying the PHA recovery and reducing the cost of downstream processes. The most demanding operation is the desalination process for PHA recovery, which needs to be addressed as it also contributes to the overall production cost. Oil palm empty fruit bunch and gluten hydrolysates are also an important waste material available in some regions and rich in carbon as well as other nutrients. In a recent study, media, nutrient, and culture optimization using these substrates as feed for *Halomonas boliviensis* resulted in a maximum of 35.7% PHA accumulation [33]. Another related species, *H. campaniensis* LS21, has the ability to secrete hydrolytic enzymes, and thus is suitable for utilizing mixed kitchen wastes as substrates for P(3HB) production. However, the yield obtained by this strain was uncompetitive with other strains, as it produced 26% P(3HB) on the mixed substrate. Expression of the PHA biosynthesis operon in this strain resulted in more than 2-fold increase in P(3HB) yield under the same cultivation condition [39]. Lignocellulosic hydrolysates are also a common source of cheap organic carbon. The potentials of the halophilic bacterium *Halomonas halophila* and the thermophilic bacterium *Schlegelella thermodepolymerans* for producing PHAs were tested using model media that mimic lignocellulose hydrolysates [44]. When provided with hexose-rich media, *H. halophila* achieved notably higher PHA yields, while *S. thermodepolymerans* exhibited a preference for media abundant in pentoses. Both extremophilic bacteria displayed higher sensitivity to microbial inhibitors compared with the mesophilic strain of *Burkholderia sacchari*. Nevertheless, taking into account their significantly enhanced PHA productivity, even in the presence of microbial inhibitors, as well as other advantageous characteristics associated with extremophiles, such as reduced susceptibility to microbial contamination, both *H. halophila* and *S. thermodepolymerans* emerge as promising candidates for sustainable PHA production. This sustainable approach capitalizes on readily available and cost-effective lignocellulosic resources. Volatile fatty acids (VFAs) are also generated from biomass, especially after anaerobic digestion or similar treatments. These VFAs are direct precursors for PHA monomers and are considered to be economical and environmentally friendly feedstocks for PHA copolymer production. However, not all the microbial strains can effectively consume large quantities of these substrates, while their higher concentration in the feed medium imposes toxicity for the cell. Very recently, the halophilic bacteria *Salinivibrio* spp. TGB4 and TGB19 were found to grow considerably on acetate or butyrate as feed (even at higher doses of up to 100 g/L). When both acetate and butyrate were fed, P(3HB) production was found to be 6.14 and 6.84 g/L for TGB4 and TGB19, respectively. Under optimum conditions, TGB19 produced a P(3HB) titer of 8.42 g/L (about 88.55% of CDM). This was further improved via fed-batch cultivation, and the P(3HB) titer reached 53.23 g/L by TGB19. Thus, P(3HB) production on VFA as substrate can be a promising way to use effluents from anaerobic digesters for large-scale plants [45]. On a similar theme, a high-concentration propionate-utilizing halophile was identified as *Halomonas* sp. YJ01, producing P(3HB-*co*-3HV) copolymer via a propionate-dependent pathway. Whole-genome analysis revealed multiple genes related to PHA biosynthesis. With 15 g/L of propionate alone, the *Halomonas* sp. was able to produce up to 29 mol% of HV content, while supplementation of glucose decreased the copolymer yield. This suggests that propionyl-CoA conversion to pyruvate must have occurred through 2-methylcitrate cycle that reduced propionate detoxification [46]. On the other hand, another thermophilic bacteria, *Caldimonas taiwanensis*, converted cassava starch to produce the biopolymer under nitrogen-limited conditions. Supplementation of a related carbon source such as valerate led to the copolymer production with a maximum PHA yield of 67% of CDM [40].

Two halophilic bacteria, *Bacillus cereus* LB7 and *Burkholderia gladioli* 2S4R1, were recently found to use mixed sugars obtained from paddy straw hydrolysis. Under optimal C:N ratios of 30:1 and 38:1 and unique growth media, PHB productivities of 0.39 g/L/h and 0.31 g/L/h, respectively [47], were achieved. This work is a unique demonstration showing bioconversion of paddy straw hydrolysate by halophilic bacteria that must be explored further for industrial-scale applications.

With the prevailing knowledge on PHA production using various microbes and feed materials at different operation scales, the success and sustainability of PHA production is now being recommended based on the concept of a circular economy. This model relies on the principle of reduce, reuse, and recycle to achieve a sustainable environment and conserve natural resources for the coming generations. From a bioprocess viewpoint, it emphasizes maximizing substrate utilization, product recovery, and energy efficiency with minimal waste generation. Compared to a linear economy, a circular economy suggests a closed-loop biorefinery design where all bio-based building blocks obtained from biomass are fractionated and converted into multiple products such as bio-fuels, PHAs, organic acids, etc. As these products are of biological origin, their usage in day-to-day life has environmental and socio-economic benefits. Here, the focus is to recycle and reuse the end-life phases for another value chain using technical solutions, modified manufacturing processes, and revised business models to achieve zero waste emission. For instance, during the PHA production process, high quantities of protein-rich effluents are generated as liquid stream. This may amount to up to 50% of the total CDM and may be collected and recovered for other suitable applications such as animal feeds, biocatalysts, or sources of amino acids [48]. There are various co-production strategies available for the simultaneous PHAs and other high-value chemicals that can be explored with integrated biorefineries for successful and sustainable PHA production in practice [49]. Thus, a “cradle-to-cradle” process is important to achieve the targets of a circular economy. The PHA polymers being biodegradable with unique physicochemical properties fits well in the concept of a circular economy. Realization of the circular economy approach on PHA production largely depends on the integration of a lignocellulosic biorefinery with unique green technologies [11,50]. It is expected to mitigate the global challenges of fossil-based fuels and polymers. Thus, factors such as the cost of pretreatment, sugars and inhibitor contents in the hydrolysates, PHA productivity, consistent polymer properties, and life-cycle and techno-economic assessment data are of importance for industrial-scale PHA production using lignocellulosic biomass. For large-scale operations of such integrated process, higher economic investment, technological advancement, and financial subsidies provided by government agencies are required [51].

## 4. Properties of PHA Produced from Waste Substrates by Extremophiles

The characterization of crucial features of PHAs is important for the determination of a suitable range of applications for which they can be processed. PHA properties are dependent on the extremophilic producer, the culture mode and conditions, and the carbon source used. P(3HB) is the most common polyhydroxyalkanoate that may be produced in a biological process by bacteria. It is highly crystalline, brittle, and stiff, with a melting temperature close to the temperature of degradation that hampers its potential application [41]. The PHA properties can be improved by incorporation of other monomeric fractions in the PHA structure. So far, not many studies have been focused on the detailed characterization of the properties of PHAs synthesized by extremophilic bacteria (Table 2). Although mcl-PHAs have better properties than scl-PHAs, there is no study that has evaluated the properties of these biopolymers produced by bacteria from extreme niches.

Dubey and Mishra (2021) reported that the M_w_ and M_n_ for the P(3HB) homopolymer produced by *H. daqingensis* grown on algal biodiesel waste residue were 309.0 and 169.8 kDa, respectively [54]. The authors observed that the thermal degradation of this biopolyester occurred at 290 °C, which is higher than that determined for commercial P(3HB). However, it was suggested that the increase in T_d_ value could be caused by impurities from the carbon source used. Lower decomposition temperature (269 °C) was observed in P(3HB) extracted from *H. alkalicola* M2 grown on bamboo powder [55]. Also, other thermal parameters such as glass and melting transition temperature are essential to determine in-service application of PHAs. Elain et al. [56] suggested that both of these factors depended on the nature of the culture medium. It was speculated that the difference in the T_m_ value may be caused by the difference in the crystallinity degree, which is significantly higher for P(3HB) produced in the culture supplemented with fruit processing water (FPW) (T_m_ = 172.4 °C for 68.3% of crystallinity) in comparison with the homopolymer synthesized from leguminous processing water (LPW) (T_m_ = 166.9 °C for 46.9% of crystallinity). Moreover, P(3HB) from the latter carbon source was characterized to have a lower T_g_ value, −5.0 °C compared to −0.2 °C for FPW.

Interestingly, it was also proven that NaCl concentration affected the molecular weight of PHAs extracted from halophilic bacteria. Pernicova et al. [32] observed that the higher the salinity applied, the lower the M_W_ value of PHB extracted from *H. hydrothermalis* cells grown on waste frying oil. However, the same authors claimed that this correlation seems to be dependent on the bacterial species. The fluctuations in the M_W_ value of PHB from *H. neptunia* were determined at various NaCl concentrations. Furthermore, Pernicova et al. [32] showed that some precursors of the 3HV fraction also influenced M_w_ in comparison with the control culture (with waste frying oil, without precursors). The molecular weight of P(3HB-*co*-3HV) copolymer extracted from *H. hydrothermalis* reached the highest value when valerate was used as the 3HV precursor. The same precursor was used by Lemechko et al. [58] in the cultivation of *Halomonas* sp. SF2003. The authors reported that the high content of the 3HV fraction (35 mol%) was associated with the carbohydrate composition of the agro-industrial effluent used as a carbon source. Moreover, the M_W_ and M_n_ values for the produced P(3HB-*co*-3HV) copolymer reached 536.8 and 389.0 kDa, respectively. Furthermore, the authors observed that the thermal properties were changeable according to the amount of 3HV monomer. It was proven that the glass transition temperature of the P(65% 3HB-*co*-35% 3HV) copolymer was the lowest (−11 °C), suggesting that the molecular mobility of the polymer chain was improved by the incorporation of a high proportion of 3HV. However, the melting temperature was not high and reached only 149.7 °C. A lower T_m_ value (139.0 °C) and higher T_g_ value (−4.7 °C) were reported for P(75.3% 3HB-*co*-24.7% 3HV) extracted from the cultivation of *Salinivibrio* sp. M318 grown on waste fish oil, glycerol, and sodium valerate [31]. However, the molecular weight was comparable to that reported for the copolymer extracted from *Halomonas* sp. SF2003 cells [58]. Interestingly, the thermal properties of P(3HB-*co*-3HV) extracted from the cultivation of *Paracoccus homiensis* grown on different renewable carbon sources did not change with the variation of 3HV [37,38]. Even though the 3HV fraction detected in the copolymer biofilm from the culture with cheese whey mother liquor was higher (60.59 mol %) than with carboxylic acids-rich stream (1.7 mol %), the T_m_ and T_g_ were comparable.

Nevertheless, there is a scarcity of data regarding the mechanical properties of PHAs extracted from the cells of extremophilic bacteria. Kim at al. [52] demonstrated that the tensile strength of P(3HB) extracted from *Halomonas* sp. YLGW01 cultured on crude glycerol was slightly lower compared with the standard. Furthermore, the Young’s modulus was detected to be much lower for the produced homopolymer (438 MPa) than for a standard P(3HB) film (1971 MPa). On the basis of these results, the authors suggested that the extremophilic producer was able to synthesize flexible biopolymer. Also, the parameter described the degree of the biofilm elongation reached the higher value (4.8%) compared to the standard (3.2%), indicating that it was less brittle.

## 5. Strategies to Enhance PHA Production from Renewable Feedstocks

### 5.1. Cultivation Approach

**Figure 3:**
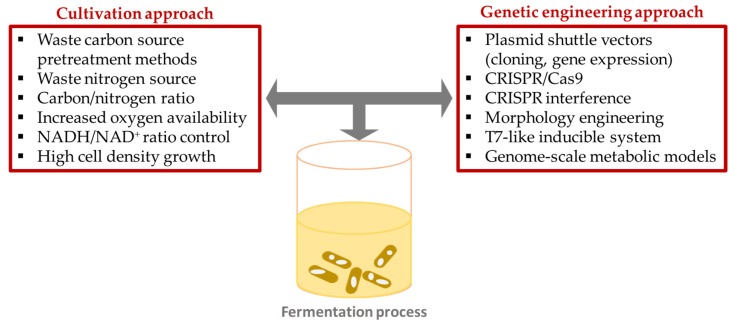
Cultivation and genetic engineering approaches to enhance PHA production.

Many key factors could affect bacterial cell metabolism, and in consequence, PHA productivity. In the past years, the operating conditions such as carbon-to-nitrogen ratio, carbon-to-phosphate ratio, carbon source concentration, temperature, or pH have been optimized to enhance PHA production (Table 3; Figure 3).

Nutrients such as carbon and nitrogen play a crucial role in regulating metabolism for optimal bacterial growth conditions [60]. In order to improve the PHA productivity, the reduction in the cost of nitrogen sources is equally desirable. Van Thuoc et al. [31] proposed to supplement the culture medium with fish sauce as a new opportunity to produce PHAs from a renewable nitrogen source and reduce biopolymer production cost as well. The authors reported that fish sauce supported *Salinivibrio* sp. M318 growth and P(3HB) production. When nitrate salts like KNO_3_ and NaNO_3_ were added to the culture as sources of nitrogen, a high P(3HB) concentration was determined. Using fish sauce and NaNO_3_ as nitrogen sources, *Salinivibrio* sp. M318 produced the highest concentration of CDM (10 g/L) and P(3HB) content (51.7% of CDM). Fish sauce was shown to be the most effective nitrogen source among the investigated sources for both bacterial growth and P(3HB) synthesis, resulting in a high P(3HB) concentration of 5.2 g/L. Additionally, the authors noted that high-quality fish sauce is not required for *Salinivibrio* sp. M318 growth; hence, fish sauce for the production of these homopolymers can be generated from fish solid waste, thereby adding value to the waste and lowering costs.

**Table 3:** Cultivation approach strategies to enhance PHA production by extremophilic bacteria.

Bacteria	Carbon Source	Cultivation Mode and Extremophile Conditions	PHA Concentration (g/L)	References
NaCl concentration				
*Salinivibrio* sp. M318	Mixture of waste fish oil and glycerol	Fed-batch bioreactor cultivation; 30 g/L NaCl	5.17	[31]
*Salinivibrio* sp. TGB10	Mixture of waste fish oil and glycerol	Fed-batch bioreactor cultivation; 27.5 g/L NaCl	27.36	[61]
Effect of waste additives				
*Yangia* sp. ND199	Crude glycerol	Fed-batch cultivations in shaking flasks; 45 g/L NaCl	8.3	[62]
	Crude glycerol + fructose corn syrup		20.3	
Effect of waste feedstock dilution				
*Halomonas halophila*	Hydrolyzed spent coffee ground	Batch cultivation; 66 g/L NaCl	2.17	[14]
	Hydrolyzed spent coffee ground 2× diluted		0.27	
	Hydrolyzed corn stover		nd	
	Hydrolyzed corn stover 2× diluted		0.82	
Effect of genetic engineering				
*Halomonas campaniensis* LS21 wild type	Alkaline seawater	Open fed continuous cultivation; 27 g/L NaCl	0.91	[39]
*Halomonas campaniensis* LS21 recombinant	Alkaline seawater	Open fed continuous cultivation; 27 g/L NaCl	2.77	
Waste feedstock treatment				
*Halomonas* sp. YLGW01	Activated carbon–treated crude glycerol	Fed-batch fermentation; C/N ratio of 10:1 (%) (*v*/*v*)	10.5	[52]
	Activated carbon–non treated crude glycerol	Fed-batch fermentation; C/N ratio of 10:1 (%) (*v*/*v*)	8.0	

nd—not detected.

Furthermore, taking into account that different inhibitors present in the substrate could hamper the bacterial cell growth, Kim et al. [52] applied activated-carbon-treated crude glycerol to minimize the negative effect of such substances. The authors conducted fed-batch fermentation of *Halomonas* sp. YLGW01 for approximately 6 d, and the first and second feedings were performed after 40 h and 64 h, respectively. However, after the first and second feeding, the crude glycerol consumption rate of the activated carbon treated group was higher than that of the control group. The concentration of inhibitors in the crude glycerol may have caused the variation in the rate of crude glycerol consumption following feeding. It is believed that MONG and ash were adsorbed on the activated carbon, even though it was not able to qualitatively confirm which inhibitors were adsorbed in the crude glycerol. These findings suggested that the adsorption of several unidentified inhibitors in crude glycerol may benefit greatly from the addition of activated carbon.

### 5.2. Genetic Engineering Approach

To construct extremophilic cells for cost-effective PHA production, new molecular approaches and techniques have been developed. The availability of tools for metabolic and morphology modifications allows the possibility of developing cell factories for extremophilic bacteria to produce various PHAs. Recently, several genome-editing tools, including CRISPR/Cas9, CRISPRi, homologous recombination, and RNA-based regulatory tools, have been proposed to enhance PHA production by *Halomonas* spp. [63]. Moreover, a number of metabolic engineering approaches have been proposed to enhance the production capability of extremophilic bacteria (Table 4; Figure 3).

Several studies were focused on the enhancement of P(3HB) production by genetically recombined extremophiles. However, none of them underlined the principles behind metabolic and genetic engineering to improve PHA production by extremophiles in the cultivations supplemented with waste feedstocks. Therefore, below, we highlight that genetic intervention in extremophilic strains has resulted in increased PHA synthesis. There are many possible ways that could be implemented to increase PHA production by extremophilic bacteria on converted renewable substrates.

*H. campaniensis* LS21 was genetically modified by an overexpression of *phaCAB* genes. Yue et al. [39] proved that the recombinant strain was able to produce higher levels of P(3HB) (70% of CDM) compared with the wild strain that synthesized only 26% of CDM during the 65-day fermentation process. Importantly, the engineered LS21 was also found to stably maintain the *phaCAB* plasmid over the entire bioprocess grown on in artificial seawater containing mixed substrates mimicking kitchen wastes. The same bacterial strain was successfully engineered to self-flocculate by deleting the *etf* gene responsible for electron transferring flavoproteins in the electron transfer chain [69]. It was reported that the *H. campaniensis* LS21 cells flocculated to the bottom of the bioreactor within 1 min after stopping of the aeration and agitation. Importantly, it was proven that the fermentation medium could be recycled without sterilization or inoculation for the growth of the next batch after collecting the precipitated cell mass.

Zhao et al. [66] proposed novel phage-derived expression systems for transcriptional control in *Halomonas* spp. TD01. The authors proved that the expression of the *phaCAB* gene, after IPTG induction, involved in P(3HB) synthesis and accumulation enhances this homopolymer content up to 92% of CDM. Moreover, the data from the literature show that redox cofactors are crucial for PHAs production [70]. It was found that *H. bluephagenesis* utilizes NADH instead of NADPH as a cofactor for P(3HB) production; thus the NADH/NAD^+^ ratio was engineered to enhance the biopolymer synthesis. Ling et al. [69] blocked an electron transfer flavoprotein pathway to increase the NADH supply, reaching up to 90% P(3HB) in comparison to 84% in cultivation of the wild type. Also, bacterial cell size and shape were changed, allowing better PHA productivity [71,72]. The overexpression of *minCD* genes, involved in cell division, resulted in the elongation of *Halomonas* sp. TD08 shape at least 1.4-fold compared with its original size, enhancing P(3HB) content from 69 to 82% of CDM in the elongated cells [2].

Extremophilic bacteria were reported to be successfully engineered to produce controllable content of 3HV fraction in P(3HB-*co*-3HV) copolymer. Clustered regularly interspaced short palindromic repeat interference (CRISPRi) has provided an efficient approach to regulate expressions of *prpC* gene encoding 2-methylcitrate synthase in *Halomonas* sp. TD01 cells. The mutant was able to produce the copolymer with regulating 3HV monomer ratio ranging from less than 1% to 13% [73]. P(3HB-*co*-4HB) copolymer is the most desirable polymer expected to be used in a wide range of applications. *H. bluephagensis* knockout was constructed to interrelate 4-hydroxybutyrate (4HB) biosynthesis pathways and guarantee a 4HB monomer supply for P(3HB-*co*-4HB) synthesis. The mutant was capable of producing 26.3 g/L of P(3HB-*co*-4HB). Moreover, the data showed that 4HB monomer content in the copolymer can be changed from 13 mol % to 25 mol % by adjusting the residual glucose concentration in the fermentation process [74].

Further studies should be conducted to understand the genetic background of extremophilic bacteria with the ability to produce PHAs, their metabolic pathways, and the possibility of enzyme modification to improve the efficiency of PHA production.

## 6. Challenges of PHA Production by Extremophiles from Waste Carbon Sources

**Figure 4:**
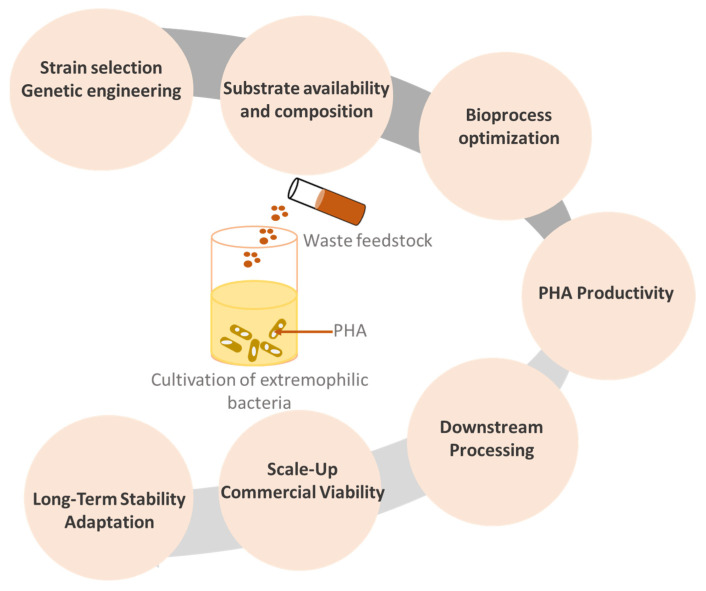
Challenges of PHA production by extremophilic bacteria from waste carbon sources.

PHA production by extremophilic bacteria from renewable resources poses several challenges that still hamper the commercial success of these bioproducts (Figure 4).

Identifying extremophilic bacteria capable of producing PHAs efficiently from waste carbon sources is a challengeable task. Numerous bacteria have the ability to produce PHAs, but in low concentrations. Once an efficient PHA producer is selected, there is a need to find the optimal conditions that both promote microbial growth and PHA accumulation, which can be also challenging. PHA production involves balancing various factors such as nutrient availability, pH, temperature, and aeration. Bacteria from harsh environments may require unique cultivation conditions due to their inherent preferences for extreme living conditions.

Moreover, the development of technologies to produce PHAs from renewable resources by extremophiles can be more demanding than the production of their counterparts. Waste management and the accessibility of feedstocks used as carbon sources should be taken into consideration as the crucial factors in this bioprocess. Extremophiles might have specific substrate preferences, and the waste feedstocks may contain impurities or inhibitory compounds that affect microbial growth and PHA accumulation. Furthermore, many agricultural wastes and bio-wastes need to be pre-treated in a manner suitable for selected bacteria.

Extremophilic bacteria could have lower growth rates and may produce PHA in lower concentrations compared with those of mesophiles. The lower PHA productivity forces enhancement of the efficiency of the bioprocess using a cultivation and genetic engineering approach. Most bacteria are not able to grow in high cell density processes. Therefore, the transition of PHA production from lab scale to industrial scale could be challenging. Ensuring consistent and high yields of PHA while maintaining the extremophiles’ preferred growth conditions requires detailed knowledge of metabolic pathways. Also, the long-term stability of PHA-producing extremophilic bacteria is crucial for consistent production. Some bacteria from extreme niches might undergo genetic changes or adaptations over time that could influence on their PHA production capabilities.

Using extremophilic bacteria for PHA production from renewable resources offers unique advantages; nevertheless, it also presents substantial challenges related to strain selection, cultivation, process optimization, productivity, and commercial viability. Overcoming these challenges will require a multidisciplinary approach involving microbiology, genetics, bioprocessing, and environmental science.

## 7. Future Perspectives

By harnessing the unique properties of extremophiles, researchers hope to develop innovative biotechnological applications that can address environmental challenges, improve industrial processes, and advance our understanding of life in extreme conditions. Extremophilic bacteria have potential to be commercialized for PHA production. However, there is still a need for the detailed analyses of the produced biopolyesters. Moreover, only a few extremophilic microorganisms have been characterized and their complete genomes sequenced. Data from the literature suggest that bacteria from extreme niches possess unique physiology and genetic and adaptation mechanisms, so it is worthwhile to study them to get insight into the regulatory genes and proteins involved in PHA biosynthesis process. It is known that PHA granules play a crucial role in adaptation to the extreme environment. To better understand the complexity of the adaptation mechanism and regulatory networks that drive PHA production omics technology (transcriptomic, proteomic and metabolomic) should be applied. Moreover, some developed systems should be tested in analyzed extremophilic bacteria, for example, a T7-like expression system was proven to benefit metabolic engineering in other non-model organisms [66].

Due to the fact that most extremophiles are predicted to be tolerable to more than one environmental factor, the classification should be re-organized considering all extremes that affect their growth and metabolism. There is still a need to conduct more comparative studies regarding the PHA production process from renewable resources to the final product. Techno-economical analysis of the PHA upstream and downstream processes should be made for extremophilic bacteria based on their abilities to tolerate specific environmental extremes [75].

## 8. Conclusions

Polyhydoxyalkanoates are natural polyesters that are produced by various microbes under specific growth conditions. For economic production of large-scale PHA production, extremophiles are considered as suitable microbial factories, and a cost-competitive platform due to less chance of mesophilic contamination and the ability to grow at high cell density. There is a growing focus on enhancing their adaptable cellular systems through synthetic biology in order to showcase a fresh biomanufacturing approach in continuous and open processes, leading to substantial reductions in costs related to process intricacies, energy consumption, substrate usage, and freshwater consumption. There is still a lack of comparison of the above-mentioned processes to the conventional ones; therefore, it is difficult to understand this bioprocesses in terms of circular bioeconomy. The development of bioreactors that can be used to cultivate bacteria under extreme conditions with the possibility to control important parameters like optical density, pH, or temperature is a crucial factor. Moreover, taking into account the economical point of view of the above-mentioned processes, there is a need to conduct research concerning the reuse of waste streams generated by the PHA production process. Through an integrative approach with lignocellulosic biorefinery, diverse products can be produced with efficient valorization of wastes. Operations with lignocellulosic raw materials at large-scales is recommended for reduction in PHA production costs and the generation of other co-products. In this context, use of extremophiles will further reduce the operational cost owing to their unique physiological requirements. Thus, the future of a PHA-based circular economy looks promising and sustainable in lieu of the biotechnological advancements made towards understanding extremophiles as single cell factories.

Furthermore, there are also some challenges that need to be overcome related to future PHA-based products. Firstly, PHAs’ low thermal stability makes them difficult to process using standard polymer processing tools, such as extruders, injection molding machines, and 3-D printers. PHAs undergo rapid and significant molecular weight reductions, color changes, and the loss of their final mechanical characteristics when it degrades thermally. Secondly, an issue with the rapid biodegradation of PHAs could hamper their commercialization in particular applications. PHA-based products still face gaps and questions that should be taken into account.

## Figures and Tables

**Table 1:** Production of PHAs by extremophilic bacteria using various renewable substrates.

Carbon Source	Microorganism	PHA Types	PHA Yield (% CDM)	Fermentation Condition	Remarks	References
Waste oils						
Palm oil mill effluent	*Salinivibrio* sp.	P(3HB)	nd	15% (*v*/*v*) POME, 5% (*w*/*v*) NaCl, 0.1% (*w*/*v*) yeast, and 0.1% (*w*/*v*) ammonium sulfate.	P(3HB) with high thermal stability	[29]
Palm oil mill effluent	*Bacillus licheniformis* M2–12	P(3HB)	88.7	pH 7, 45 °C	PHA production using cheap raw materials	[30]
Waste fish oil and glycerol	*Salinivibrio* sp. M318	P(3HB-*co*-3HV)	51.5	pH 6.5, 30 °C, 0.5 g/L KH_2_PO_4_, 600 rpm	Higher biomass of 69.1 g/L was obtained after 78 h in fed -batch culture.	[31]
Waste frying oil	*Halomonas hydrothermalis*	P(3HB)	61.98	pH 7.5, 30 °C, 40 g/L NaCl, 300 rpm	Supplementation of valerate led to an HV content of 50.15 mol%	[32]
	*Halomonas neptunia*	P(3HB)	55.71	pH 7.5, 30 °C, 60 g/L NaCl, 300 rpm	Supplementation of n-propanol led to an HV content of 29.5 mol%	
Waste cooking oil	*Paracoccus* sp. LL1	P(3HB-*co*-3HV)	30.89	pH 7.5, 30 °C, 1.0 g/L (NH_4_)_2_SO_4_, 0.1% Tween-80, 300 rpm, 2.5 vvm	Batch culture with 3.24 g/L biomass and 0.89 mg/L carotenoids	[16]
Oil Palm Empty Fruit Bunch	*Halomonas boliviensis*	P(3HB)	35.7	pH 7, 31 °C, 2.5 g/L KH_2_PO_4_, 200 rpm	Non-conventional nutrients for cost-effective PHA production	[33]
Crude glycerol						
Biodiesel fuel by-product	*Thermotolerant Pseudomonas* sp. strain SG4502	mcl-PHA	40.6	45 °C, minimal salt media, 160 rpm	PHA production at high temperature	[34]
Jatropha biodiesel byproduct	*Halomonas hydrothermalis* SM-P-3M	P(3HB)	75.0	pH 7, 37 °C, 0.4 g/L KH_2_PO_4_, 200 rpm	High PHB content by a marine environment isolate	[35]
Crude glycerol	*Paracoccus* sp. LL1	P(3HB-*co*-3HV)	39.3	pH 7.5, 30 °C, 1.0 g/L (NH_4_)_2_SO_4_, 300 rpm	Cell-retention culture with 24.2 g/L of biomass and 7.14 mg/L of carotenoids	[15]
Residues from cheese production						
Whey-based media	*Thermus thermophilus* HB8	P(3HB-*co*-3HHp-*co*-3HN-*co*-3HU)	35	initial phosphate concentration of 50 mM	Novel heteropolymer consisting of both *scl*- and *mcl*-PHA	[36]
Cheese whey mother liquor	*Paracoccus homiensis*	P(3HB-*co*-3HV)	29.0	pH 7.6, 28 °C, 3.0 g/L KH_2_PO_4_, 110 rpm	Utilization and management of dairy wastes	[37]
Acidogenic fermentate of acid whey			17.0	pH 5.5, 30 °C, UASB reactor with autocontrols	Valorization of carboxylic acid rich waste streams	[38]
Other waste materials						
Mixed substrates (Kitchen wastes)	*Halomonas campaniensis* strain LS21	P(3HB)	26	27 g/L NaCl, pH 10, 37 °C for 65 days	Secreted extracellular enzymes for waste hydrolysis	[39]
	Recombinant *H. campaniensis*	P(3HB)	70		Secreted enzymes and also maintained the *phbCAB* plasmid throughout the fermentation process without contamination	
Cassava starch + valerate	*Caldimonas Taiwanensis*	P(3HB-*co*-3HV)	67	Nitrogen limited conditions (C/N = 30)	PHA production by thermophilic bacteria	[40]

CDM: Cell dry mass; P(3HB)—poly(3-hydroxybutyrate); P(3HB-*co*-3HV)—poly(3-hydrobutyrate-*co*-3-hydrovalerate) copolymer; P(3HB-*co*-3HHp-*co*-3HN-*co*-3HU)—poly (3-hydroxyvalerate-*co*-3-hydroxyheptanoate-*co*-3-hydroxynanoate-*co*-3-hydroxyundecanoate) copolymer.

**Table 2:** Material properties of PHAs extracted from extremophilic bacteria.

Bacteria	Carbon Source	Type of PHA	M_w_ (kDa)	M_n_ (kDa)	T_m_ (°C)	T_g_ (°C)	T_d_ (°C)	References
*Halomonas* sp. *YLGW01*	Crude glycerol	P(3HB)	580.0	430.0	nd	nd	nd	[52]
*Halomonas elongata P2*	Wheat straw	P(3HB)	nd	nd	165.0	nd	nd	[53]
*Halomonas daqingensis*	Algal biodiesel waste residue	P(3HB)	309.0	169.8	nd	nd	290.0	[54]
*Halomonas ventosae*	Algal biodiesel waste residue	P(3HB)	nd	nd	nd	nd	296.0	[54]
*Halomonas hydrothermalis*	Waste frying oil	P(3HB)	253.6	216.8	nd	nd	nd	[32]
*Halomonas alkalicola M2*	Bamboo powder	P(3HB)	390.0	613.0	163.0	nd	269.0	[55]
*Salinivibrio* sp. M318	Waste fish oil + glycerol	P(3HB)	410.0	300.0	170	4.0	nd	[31]
*Halomonas* sp. i4786	Leguminous Processing Water	P(3HB)	677.5	644.5	166.9	−0.2	nd	[56]
	Fruit Processing Water	P(3HB)	588.0	518.5	172.4	−5.0	nd	
*Halomonas* sp. SK5	Oil palm trunk	P(3HB)	165.0	827.0	nd	nd	nd	[57]
*Paracoccus homiensis*	Carboxylic acids-rich stream	P(98.3% HB-*co*-1.7% HV)	nd	nd	165.4 (155.0)	2.5	253.8	[38]
*Paracoccus homiensis*	Cheese whey mother liquor	P(39.41% HB-*co*-60.59% HV)	nd	nd	167.7 (158.2)	2.3	280.0	[37]
*Halomonas* sp. *SF2003*	Agro-industrial effluent/valeric acid	P(65% HB-*co*-35% HV)	536.8	389.0	149.7 (166.0)	−11.7	nd	[58]
*Halomonas campisalis MCMB-1027*	Bagasse extract	P(94.4% HB-*co*-5.6% HV)	139.4	838.5	168.9	nd	nd	[59]
*Salinivibrio* sp. M318	Waste fish oil + glycerol + sodium valerate	P(75.3% HB-*co*-24.7% 3HV)	530.0	310.0	139.0	−4.7	nd	[31]

nd—not detected; P(3HB)—poly(3-hydroxybutyrate); P(3HB-*co*-3HV)—poly(3-hydrobutyrate-*co*-3-hydrovalerate) copolymer.

**Table 4:** Genetic engineering strategies to enhance PHA production by extremophilic bacteria.

Bacteria	Genetic Engineering Strategy	Benefits	References
*Halomonas bluephagenesis* TD01	Promoter engineering	80% of P(3HB-*co*-4HB) in CDM under non-sterile fed-batch fermentation	[64]
*Halomonas* sp. KM-1	CRISPR/Cas9 system for genome deletion and integration	disruption of the *pyrF* gene	[65]
*Halomonas* sp. TD01	T7-like system for overexpression of the cell-elongation cassette (*minCD* genes)	100-fold increase in cell lengths and high levels of P(3HB) production (up to 92% of CDM)	[66]
*Halomonas bluephagenesis*	Ligand-induced system for the control of *minCD*, and monomer precursor 4-hydroxybutyrate-CoA (4HB-CoA) synthesis pathway	over 10 g/L of P(3HB) accumulated by elongated cell sizes, and 6 g/L of P(3HB-*co*-9.57 mol% 4HB) copolymer	[67]
*Halomonas campaniensis* LS21	Temperature-responsible plasmid expression system for inactivation of *mreB* and *ftsZ* genes	controllable expanding cell volumes for PHA granules, up to 80% P(3HB) yield	[68]
*Halomonas campaniensis* LS21	Deletion of the etf operon encoding two subunits of an electron transfer flavoprotein for reduction of downstream cost associated with continuous centrifugation	Most microbial cells flocculated and precipitated to the bottom of the bioreactor within 1 min after stopping the aeration and agitation.	[69]

## Data Availability

The data that support the findings of this study are available from the corresponding authors, upon reasonable request.
